# Superoxide Dismutase 3 Limits Collagen-Induced Arthritis in the Absence of Phagocyte Oxidative Burst

**DOI:** 10.1155/2012/730469

**Published:** 2012-03-05

**Authors:** Tiina Kelkka, Juha Petteri Laurila, Outi Sareila, Peter Olofsson, Mikko Olavi Laukkanen, Rikard Holmdahl

**Affiliations:** ^1^Medicity Research Laboratory, University of Turku, Tykistökatu 6 A, 20520 Turku, Finland; ^2^Turku Doctoral Programme of Biomedical Sciences, Kiinamyllynkatu 13, 20520 Turku, Finland; ^3^Medical Inflammation Research, Department of Medical Biochemistry and Biophysics, Karolinska Institutet, 171 77 Stockholm, Sweden; ^4^Redoxis AB, Sahlgrenska Science Park, Medicinaregatan 8A, 413 46 Göteborg, Sweden; ^5^Fondazione SDN, 80143 Naples, Italy

## Abstract

Extracellular superoxide dismutase (SOD3), an enzyme mediating dismutation of superoxide into hydrogen peroxide, has been shown to reduce inflammation by inhibiting macrophage migration into injured tissues. In inflamed tissues, superoxide is produced by the phagocytic NOX2 complex, which consists of the catalytic subunit NOX2 and several regulatory subunits (e.g., NCF1). To analyze whether SOD3 can regulate inflammation in the absence of functional NOX2 complex, we injected an adenoviral vector overexpressing SOD3 directly into the arthritic paws of *Ncf1^∗/∗^* mice with collagen-induced arthritis. SOD3 reduced arthritis severity in both oxidative burst-deficient *Ncf1^∗/∗^* mice and also in wild-type mice. The NOX2 complex independent anti-inflammatory effect of SOD3 was further characterized in peritonitis, and SOD3 was found to reduce macrophage infiltration independently of NOX2 complex functionality. We conclude that the SOD3-mediated anti-inflammatory effect on arthritis and peritonitis operates independently of NOX2 complex derived oxidative burst.

## 1. Introduction

Extracellular superoxide dismutase (SOD3) is an enzyme known to catalyze dismutation of the highly reactive, superoxide anion into longer-lived and more stable hydrogen peroxide [1]. The consequences of SOD3 action in the cells reach beyond the antioxidative functions, as it has been shown to downregulate inflammation [2], stimulate cell proliferation during tissue injury recovery [3], and to counteract apoptosis [4, 5] by affecting cytokine production, cell signal transduction, and expression of survival-related genes.

The anti-inflammatory properties of SOD3 have been studied in models of pulmonary disease and peritonitis [2, 6]. Previous work with collagen-induced arthritis (CIA) suggests that both genetic transfer of the SOD3 gene [7] as well as a small molecular SOD mimetic have the ability to ameliorate arthritis [8]. The arthritis ameliorating effect of SOD3 was later confirmed using SOD3 knock-out mice [9]. These results were explained on the basis that SOD3 acts as an antioxidant and catalyses dismutation of superoxide into hydrogen peroxide, thus reducing inflammation-induced oxidative stress and restoring the oxidant balance in the arthritic joints [10, 11]. This explanation, however, is difficult to reconcile with the finding that animals naturally deficient in the induced oxidative burst in fact develop more severe arthritis [12, 13].

The most potent producer of superoxide, the substrate for SOD3, is the well-characterized phagocytic NOX2 complex. In inflamed tissues NOX2 complex produces massive amounts of superoxide upon activation in a process called phagocyte oxidative burst. In addition to NOX2, superoxide is produced from various other cellular sources, such as from the mitochondria during cellular respiration and by other members of the NOX enzyme family [14]. However, it should be noted that during inflammation these superoxide producers are not nearly as efficient superoxide producers as the NOX2 complex.

In the current work we studied the role of SOD3 in collagen-induced arthritis (CIA) to understand whether the therapeutic effect of SOD3 on arthritis operates through attenuating the biological effects of the induced oxidative burst produced by the NOX2 complex. To avoid artifacts introduced by chemical inhibitors of NOX2 complex [14–16] we used mice that genetically lack functional NOX2 complex. Thus we used wild-type (*Ncf1^+/+^*) and *Ncf1* mutated (*Ncf1^∗/∗^*) mice on B10.Q background. These strains differ at only one SNP in the *Ncf1* gene, which makes the *Ncf1^∗/∗^* strain unable to produce oxidative burst [13]. The mutated mouse is more susceptible to induced arthritis due to hyperactivated T cells [13], and also increased susceptibility to thioglycollate peritonitis has been reported in the *Ncf1* knockout mouse [17]. Our results confirm the previously documented anti-inflammatory role of SOD3 and additionally, for the first time, we show that it can downregulate both CIA and peritonitis even in the absence of functional NOX2 complex and phagocyte oxidative burst.

## 2. Materials and Methods

### 2.1. Mice

The previously described *Ncf1^m1J^* (protein also called *p47phox*) mouse [18], which carries a point mutation globally and completely abolishing NOX2 complex derived ROS production, has been backcrossed onto the B10.Q background [13] and shown to contain only the causative mutation using a 10 k SNP typing chip. The mice were housed under specific pathogen-free conditions in climate-controlled environment and fed standard rodent chow and water ad libitum at Turku University Central Animal Facility. All experimental mice were sex- and age-matched, treatment groups were blinded, and experimental groups were mixed in cages in all experiments. The experiments were performed in accordance with the national and EU guidelines and the study was approved by the Oulu section of the national Animal Experiment Board (Eläinkoelautakunta, ELLA) with ethical approval numbers ESLH-2008-02873, ESLH-2008-07941, and ESAVI-0000497/041003/2011.

### 2.2. Viral Expression Vectors Used in the Study

Replication deficient adenoviral E1-partially-E3-deleted AdBglII vectors (developed from serotype Ad5) expressing rabbit SOD3 (Ade-SOD3) or bacterial *β*-galactosidase lacZ (Ade-LacZ) [4] were used in both in vitro and in vivo experiments.

### 2.3. Collagen-Induced Arthritis

Collagen-induced arthritis (CIA) was induced under isoflurane anesthesia by injecting 100 *μ*g rat type II collagen (purified from chondrosarcoma) [19] emulsified in complete Freund's adjuvant intradermally at the base of the tail. Arthritis was boosted day 19 with 50 *μ*g rat type II collagen emulsified in incomplete Freund's adjuvant intradermally at the base of the tail. Disease development was evaluated macroscopically three times a week before the booster immunization and daily after the boost [20]. One point was given for each swollen toe or joint and five points for a swollen ankle, each paw having the maximum of fifteen points.

In the arthritis experiments Ade-SOD3, Ade-LacZ (vector control), and PBS (injection control) were injected locally in the left front paw in 25 *μ*L injection volume containing 2,5 × 10^8^ PFU virus. Injections were performed right after the booster immunization during the same anesthesia at day 19, before the onset of clinically apparent arthritis.

### 2.4. Peritonitis

Peritonitis experiments were performed as described in [2]. Briefly, mice were pretreated i.p. with 0.5 × 10^9^ PFU Ade-SOD3, Ade-LacZ, or PBS three days before peritonitis induction with 5% proteose peptone (BD Difco, Sparks, MD, USA) and 10 ng IL-1*β* (R&D Systems, Minneapolis, MN, USA) in 1 mL PBS. After 18 hours peritoneal infiltrating cells were collected with 10 mL ice-cold RPMI cell culture medium. Cells from the peritoneal lavage were counted and cytocentrifuged, slides were stained with Reastain Diff-Quick (Reagena, Toivala, Finland), and differential counting was performed under a standard light microscope. Experiments were pooled and the total cell numbers are presented as percentual increase from the PBS injection control. All peritonitis results were normalized to adjust the vector control group (Ade-LacZ) mean to 100.

### 2.5. Cell Culture and In Vitro Extracellular Oxidative Burst

COS-7 cells stably expressing all the essential components of the NOX2 complex, namely, Cybb (gp91phox), Cyba (p22phox), Ncf2 (p67phox), and Ncf1 (p47phox) were provided by Dr. Mary C. Dinauer, Indiana University, USA [21]. Cells were cultured in Dulbecco's complete medium (Gibco), 10% fetal calf serum, and penicillin-streptomycin (Invitrogen, Paisley UK).

Extracellular superoxide production was quantified two days after transduction (MOI 4) with adenoviral constructs (Ade-SOD3 and Ade-LacZ) or medium control directly on the 96-well cell culture plate using an isoluminol-enhanced chemiluminescence method [22, 23]. Briefly, the cells were washed with PBS, and 100 *μ*L of isoluminol buffer was added in each well. Isoluminol buffer contained isoluminol (10 *μ*g/mL, Sigma-Aldrich) and horse radish peroxidase-type II (4 U/mL, Sigma-Aldrich) dissolved in PBS with PMA (200 ng/mL, dissolved in DMSO, Sigma-Aldrich) and data collection was initiated immediately and followed at 37°C as produced luminescence signal (Tecan Infinite M200, Tecan, Männedorf, Switzerland) for 30 minutes. DMSO vehicle controls represent the nonstimulated background values. Representative data from the 15-min time point is reported.

### 2.6. In Vitro Intracellular Oxidative Burst

Red blood cells were lysed from heparinized whole blood with hypotonic lysis buffer and leukocytes were surface stained with APC conjugated anti-Gr-1 (RB6-8C5) and eFluor 450 conjugated anti-CD11b (M1/70) antibodies (eBioscience). Cells were suspended in high-glucose D-MEM (Gibco) with antibiotics without FCS and incubated for 10 min at 37°C with 3 *μ*M dihydro-rhodamine 123 (DHR-123; Molecular Probes and Invitrogen Life Technologies) followed by 20 min activation at 37°C with 200 ng/mL PMA (Sigma-Aldrich). After oxidation by ROS, DHR-123 emits fluorescence upon excitation with the blue laser. The cells were washed into PBS and acquired on LSR II flow cytometer equipped with FACS Diva software (BD Biosciences). Live cells were gated on the cell type, and geometric means of respective populations were analyzed with Flowing Software (Cell Imaging Core, University of Turku).

### 2.7. In Vivo Oxidative Burst

Isoflurane anesthetized animals with equal arthritis scores were injected intraperitoneally with 20 mg/kg L-012 probe (Wako Chemicals, Germany) dissolved in PBS [24]. The luminescent signal was detected with IVIS 50 bioluminescent system (Xenogen, USA) that consists of an anesthesia unit built in a light tight chamber equipped with a CCD camera. Image acquisition and analysis were performed with Living Image software (Xenogen).

### 2.8. Detection of Ade-SOD3 from the Injected Paws by RT-PCR

RNA was isolated from the treated paws collected d25 according to the manufacturer's instructions with TRI Reagent (Sigma-Aldrich). Glass homogenizers were used to homogenize and separate the soft tissue from the bone. The isolated RNA was DNAse treated with deoxyribonuclease I (Fermentas) in the presence of Ribolock RNase Inhibitor (Fermentas). The RNA was used for reverse transcription reaction performed with Revert-Aid M-MULV (Fermentas). The acquired cDNA was subjected to Q-PCR with Ade-SOD3 (fw: GTG TGC TCC TGC CTG CTC, rev: CTG CTC CAC CGT GTC TGA G) and *β*-actin specific primers (fw: CTA AGG CCA ACC GTG AAA AG, rev: ACC AGA GGC ATA CAG GGA CA), and the gene expression level was analyzed using SYBR Green PCR Master Mix (Applied Biosystems), iCycler iQ Multicolor Real-Time PCR Detection System (Bio-Rad), and the iCycler (version 3.1) software. Ade-SOD signal was normalized against *β*-actin expression levels and the results are reported as fold change from the Ade-LacZ group mean denoted as 1.

### 2.9. Statistics

Quantitative data are expressed as ± SEM. Statistical significance was analyzed by using two-tailed Student's *t*-test or if more than two groups were analyzed one-way ANOVA with LSD post hoc analysis was run using IBM SPSS Statistics 19 software (SPSS Inc.), *P* < 0.05 is considered as statistically significant.

## 3. Results

### 3.1. Ade-SOD3 Produced Enzymatically Active SOD3 In Vitro

**Figure 1:**
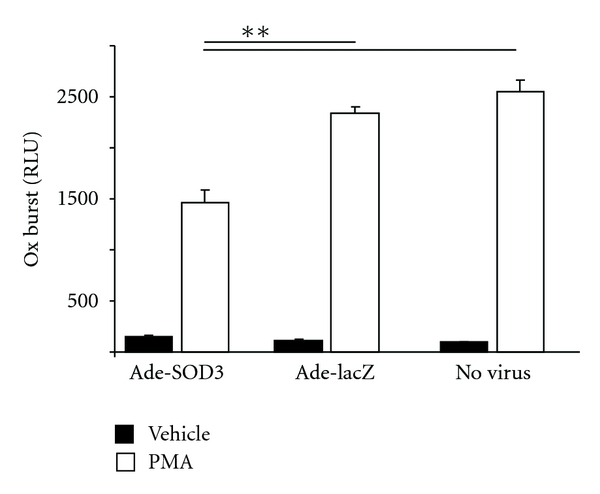
The amount of extracellular ROS is reduced by SOD3. Adenoviral vector coding for SOD3 (Ade-SOD3) significantly reduced the amount of extracellular ROS produced by COS cells expressing all functional components of the NOX2 complex, when compared to the Ade-LacZ control virus.

Adenoviral SOD3 gene delivery reduced the superoxide predominant ROS signal 37% compared to the Ade-LacZ control virus when investigated two days after transduction with Ade-SOD3 and Ade-LacZ viruses (Figure 1).

### 3.2. SOD3 Downregulated Arthritis

**Figure 2:**
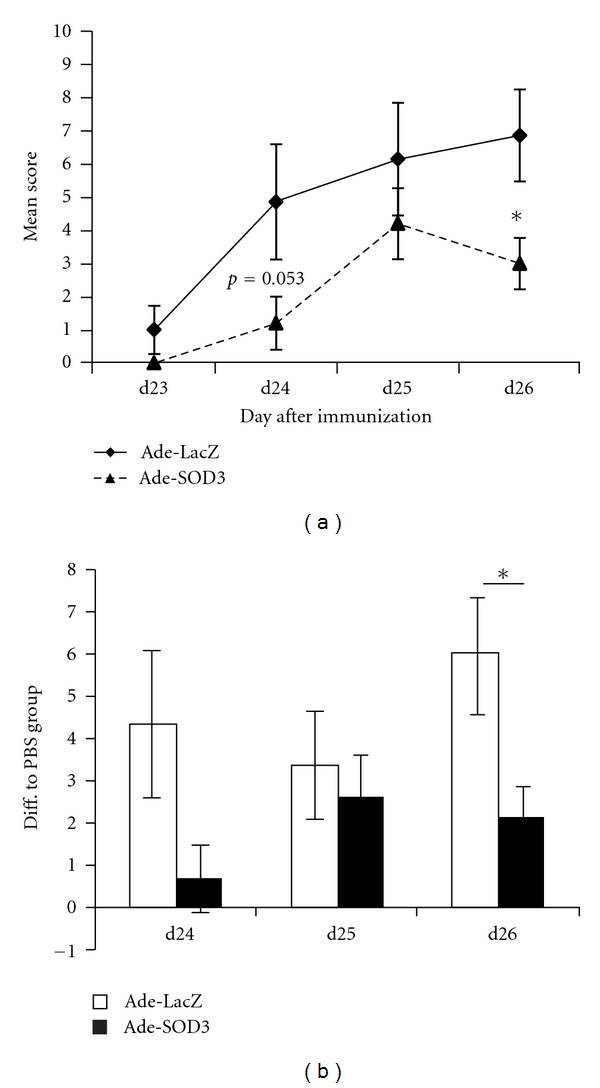
SOD3 downregulated arthritis in wild-type B10.Q mice. Adenovirally expressed SOD3 reduced arthritis severity in the treated paws (a) in wild-type B10.Q mice (Ade-SOD3 *n* = 6, Ade-lacZ *n* = 7). When compared to the PBS-treated injection control (*n* = 8) group, Ade-SOD3-treated mice had less increase in the arthritis score in the treated paw than the Ade-LacZ-treated control mice (b).

Mice started to develop mild, clinically apparent arthritis at day 20 and made a full response, mean arthritis scores reaching 7 (out of the maximum of 15) in Ade-Laz treated and 3 points in Ade-SOD3-treated mice some days later. The mean disease score in the treated paws was lower in the Ade-SOD3-treated group when compared to the Ade-LacZ-injected control group, and the difference reached statistical significance at d26 (Figure 2(a)). The treatment effect was only seen in the treated paw, while there were no differences between the treatment groups in sum scores of the three untreated paws (see Supplementary Figure 1(a) in supplementary material available online at doi: 10.1155/2012/730469) highlighting the local character of the used gene therapy vector expressing SOD3.

Both virus vector treated groups showed elevated arthritis scores in the treated paws when compared to the PBS-injected control paws in the injection control group. This difference is illustrated in Figure 2(b), where Ade-LacZ-treated paws are shown to have larger increase in arthritis score than the Ade-SOD3-treated paws.

### 3.3. SOD3 Downregulated Arthritis in the Absence of Functional NOX2 Complex

**Figure 3:**
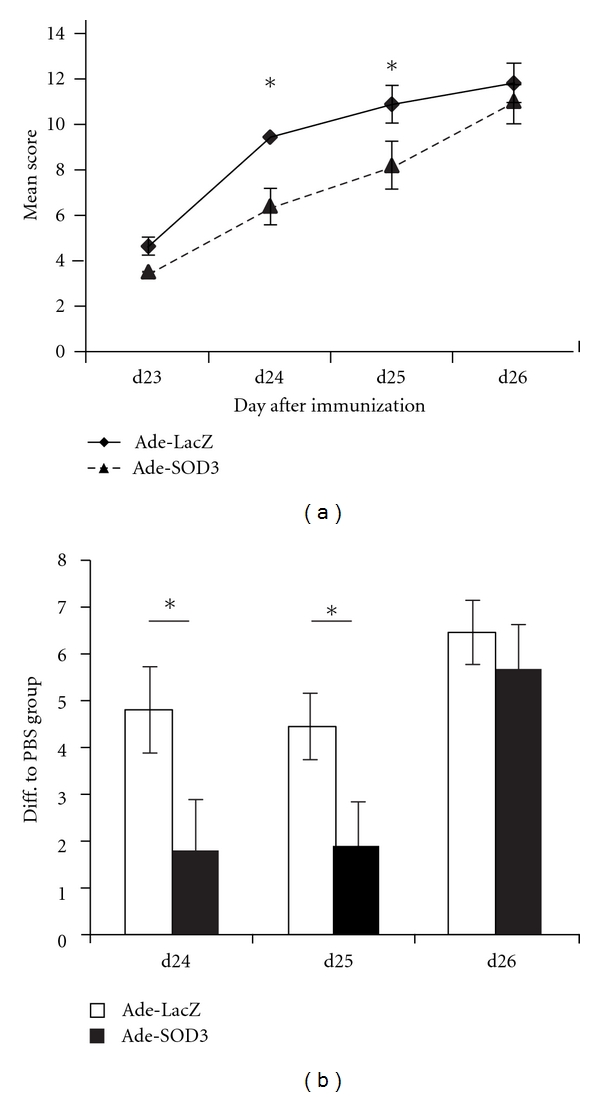
SOD3 downregulated arthritis in the absence of functional NOX2 complex. Arthritis severity was significantly reduced in Ade-SOD3-treated paws of mice lacking functional oxidative burst when compared to the control vector-treated paws (a). Shown is pooled data from two independent experiments (Ade-SOD3 *n* = 22, Ade-lacZ *n* = 23). The difference in arthritis score in the treated paw between the PBS-treated control mice (*n* = 10) and Ade-SOD3-treated mice was smaller than the difference between PBS-treated and Ade-LacZ-treated mice at days 24 and 25 (b).

Starting from d20 the *Ncf1^∗/∗^* mice started to develop arthritis with significantly higher mean score than the wild-type mice. Interestingly, the mutated mice without functional NOX2 complex derived superoxide production also responded to Ade-SOD3 treatment. Ade-SOD3-treated paws of the *Ncf1^∗/∗^* mice had significantly lower mean disease score than the Ade-LacZ-treated vector control paws at days 24 and 25 after arthritis induction. Similarly to the wild-type mice, the difference in the mean arthritis score in NOX2 complex deficient mice was observed when SOD3 was highly expressed from the adenoviral vector (Figure 3(a)) [4]. No differences were observed between the treatment groups in the sum scores of the untreated paws (Supplementary Figure 1(b)) again supporting the local character of the immunomodulatory effect of Ade-SOD3 treatment.

Similarly to the wild-type mice, Ade-SOD3 treatment in *Ncf1^∗/∗^* paws induced significantly smaller difference between the virus-treated and PBS-injected paws than Ade-LacZ virus injection (significant d24 and d25 after immunization), again confirming the arthritis limiting effect of SOD3 (Figure 3(b)). The experiment was repeated and as the experiments were well reproduced, data from both experiments was combined for analysis.

### 3.4. SOD3 Limited Peritonitis Both in the Presence and Absence of Phagocyte Oxidative Burst

**Figure 4:**
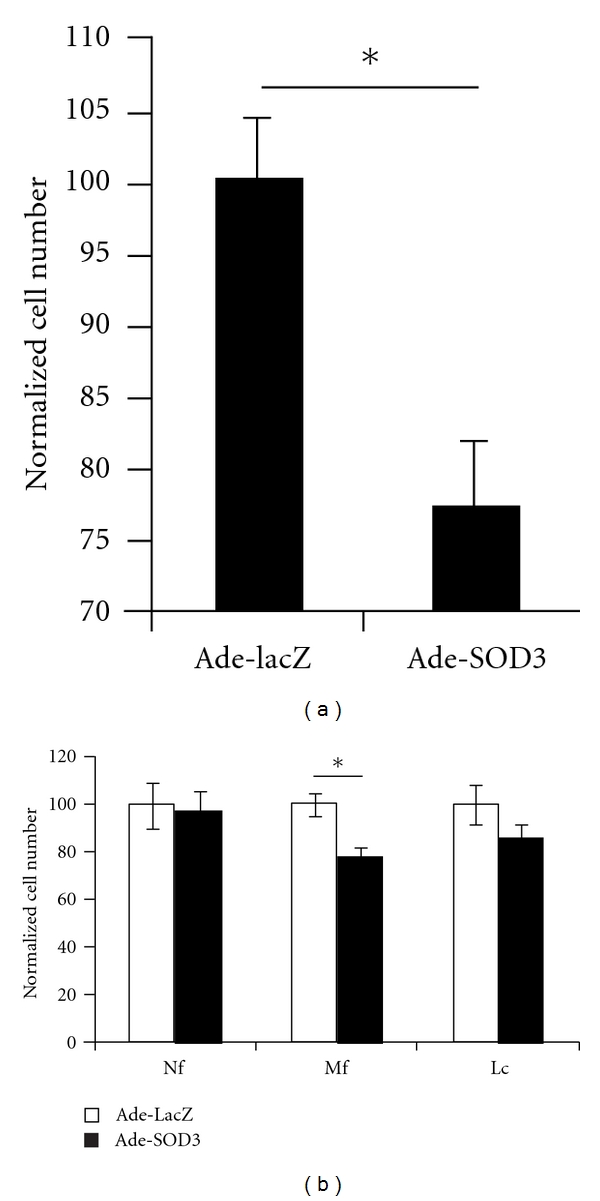
Peritonitis is limited by SOD3. Ade-SOD3-treated mice had less peritoneal infiltrating cells after peritonitis induction with proteose peptone and IL-1*β* than Ade-LacZ-treated control mice (a) (*n* = 5). The reduction in cell number was mainly due to diminished peritoneal macrophage population (b) (Nf = neutrophil, Mf = macrophage, Lc = lymphocyte).

SOD3 expression significantly reduced the number of peritoneal infiltrating cells in proteose peptone and IL-1*β* induced peritonitis in wild type mice (Figure 4(a)). The decrease in infiltrating leukocytes was mainly due to a lowered number of infiltrating macrophages (Figure 4(b)), which well corresponds with the macrophage phase of peritonitis taking place three days after virus injection and 18 hours after peritonitis induction. Both virus-treated groups had more infiltrating cells than the PBS-injected control mice.

**Figure 5:**
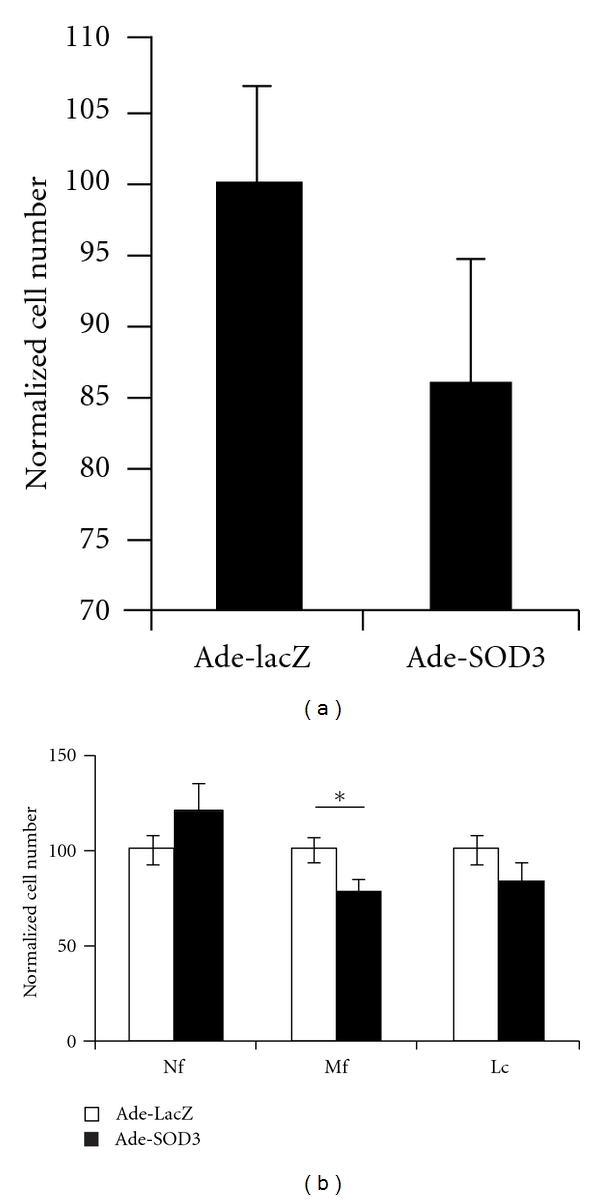
Peritonitis is limited by SOD3 in the absence of functional NOX2 complex. Similarly to wild-type mice, Ade-SOD3-treated *Ncf1^∗/∗^* mice had less peritoneal infiltrating macrophages than the Ade-LacZ-treated vector controls in the proteose peptone and IL-1*β*-induced peritonitis model (a). Normalized total cell numbers (b) show significant decrease in infiltrating macrophage population. Shown is pooled data from two independent experiments (*n* = 10) (Nf = neutrophil, Mf = macrophage, Lc = lymphocyte).

Similarly as in the wild-type mice Ade-SOD3 was shown to reduce the number of infiltrating cells in the peritoneal cavity of *Ncf1^∗/∗^* mice when compared to Ade-LacZ-treated mice. The difference was due to a significantly lowered number of infiltrating macrophages in the Ade-SOD3-treated mice (Figures 5(a) and 5(b)).

### 3.5. *Ncf*1^∗/∗^ Mice Lack Oxidative Burst In Vivo and In Vitro

**Figure 6:**
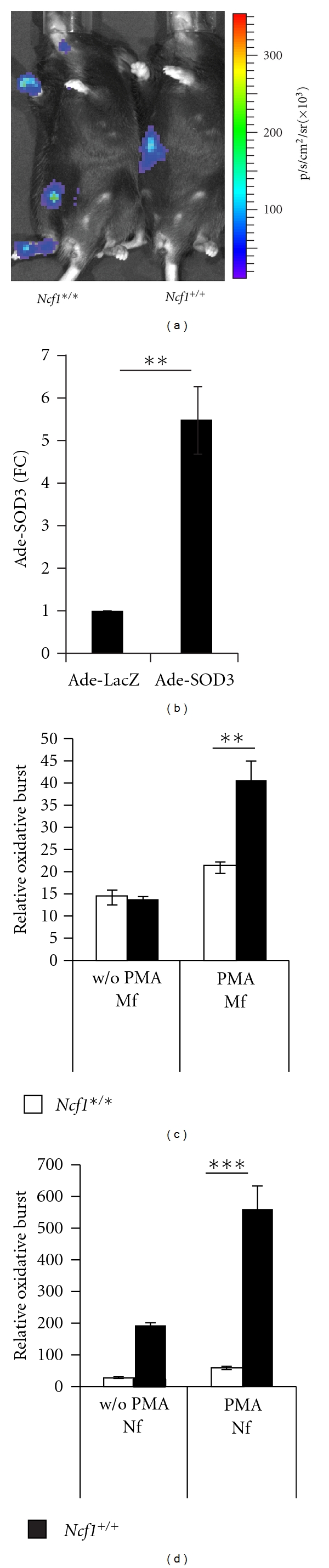
No oxidative burst was detected from the *Ncf1* mutated mice. In vivo imaging with the ROS sensitive L-012 probe revealed a bright luminescent signal from the inflamed wild-type paws with CIA, while no signal was detected from *Ncf1^∗/∗^* mice with comparable arthritis severity (a). Imaging was reproduced in two independent experiments with similar results. Expression of SOD3 by Ade-SOD3 expression vector in the treated paws was confirmed by Q-RT-PCR (*n* = 7/group) (b). Ex vivo oxidative burst was measured from phagocytes from *Ncf1^∗/∗^* (*n* = 5) and wild-type mice (*n* = 6) by using DHR-123 assay with and without (w/o) PMA stimulation. Neither macrophages (c) nor neutrophils (d) from the *Ncf1^∗/∗^* mice were able to exert a measureable oxidative burst.

In the wild-type mouse both severely inflamed paws (right hind leg and right front paw) emitted strong ROS-induced L-012 luminescence signal, while in spite of the severe inflammation, no luminescent signal was detected from the *Ncf1^∗/∗^* mice (severe arthritis with arthritis score of 15 in both front paws and milder symptoms in the left hind leg) (Figure 6(a)). The intraperitoneal L-012 injection site emitted similar background signal in both genotypes.

Monocytes (CD11b-POS, Gr-1-LO, or Gr-1-NEG) and granulocytes (Gr-1-HI, CD11b-POS) from *Ncf1^∗/∗^* mice were unable to generate efficient ROS production upon PMA stimulation, while phagocytes from the wild-type animals responded to PMA stimulation and induced significant increase in DHR-123 derived fluorescence signal detected by flow cytometry (Figures 6(c) and 6(d)).

### 3.6. Ade-SOD3 Expression In Vivo

Quantitative RT-PCR revealed a A 5.5-fold (FC, fold change) expression of adenovirally produced SOD3 mRNA in the Ade-SOD3-treated joints collected at d25 when compared to the Ade-LacZ-treated vector control paws (Figure 6(b)).

## 4. Discussion

SOD3 is an enzyme that has been shown to give rise to therapeutic responses in damaged tissues such as reduced ischemia-reperfusion injury [25], arthritis [7], peritonitis [2], hind limb injury [3], and lung injury [6] models. These tissue healing promoting and anti-inflammatory effects induced by SOD3 are accompanied by reduced macrophage infiltration [2], inhibition of oxidative fragmentation of the extracellular matrix matrix [26], decreased apoptosis [4, 5], and enhanced cell proliferation [27]. The beneficial effects of SOD3 are mostly explained by its antioxidant properties and reduction of oxidative stress in the injured tissues. However, in this report we show that overexpression of SOD3 downregulated inflammation even in the absence of phagocyte oxidative burst, thus highlighting the capacity of SOD3 to affect cellular processes independently of NOX2 complex' superoxide production.

The adenoviral gene expression system used in this work reaches its maximal expression around three days after the injection, after which the vector is eliminated by the immune system and the expression of the transgene slowly decreases to undetectable levels 14 days after the initial injection [4]. We confirmed the presence of virally delivered SOD3 mRNA in the paws d25 and thus confirmed that the decrease in arthritis severity coincided with substantial adenovirus driven SOD3 expression. Similarly, pretreating the mice three days before induction of peritonitis allowed us to analyze the effect of SOD3 during substantial SOD3 expression in the peritoneal cavity.

The percentual treatment effect in both inflammation models was comparable with the effect previously obtained with transgenic SOD3 overexpression in pulmonary emphysema [26]. Similarly, the degree of SOD3-induced macrophage infiltration inhibition in peritonitis was similar as reported previously [2]. Even though SOD3 is an important local regulator of the acute inflammatory reaction, it is obvious that there are a number of other factors affecting inflammation severity in vivo.

The physiological function of SOD3 is to dismutate extracellular superoxide into hydrogen peroxide. When the NOX2 complex is not functional, there are still a number of other enzymes and enzyme complexes producing superoxide in the inflamed tissue. NOX1 has been reported to worsen hyperoxia-induced acute lung injury in mice [28], and NOX4 has been suggested to stimulate microglial IL-6 expression [29] and to hamper neurodegeneration after poststroke ischemia reperfusion injury [30]. However, only NOX2 is abundantly expressed on phagocytes and thus recruited to inflammatory foci. Other NOX family members are expressed on other cell types than phagocytes and their ROS production is not upregulated during inflammation.

In addition to the NOX family enzymes, superoxide is also produced during mitochondrial respiration at levels corresponding to the general metabolic rate in the tissue. Mitochondrial superoxide has also been linked to TLR2/4 signaling [31] and is also suggested as a pathologic mechanism in tissue injury [32]. All these superoxide generating processes, however, cannot compensate for the massive production of ROS by the NOX2 complex during inflammation as the arthritic paws of *Ncf1^∗/∗^* mice emitted no detectable luminescence signal when probed with L-012 dye (Figure 6(a)). L-012 reacts with any radical to produce light and cannot be used to distinguish between superoxide and hydrogen peroxide in vivo.

As a tool to study the NOX2 complex dependency of SOD3, we used *Ncf1* mutated mice, in which a splice site point mutation abolishes the production of NCF1 protein leading to complete loss of NOX2 complex derived oxidative burst. In line with previous reports, arthritis severity was significantly higher in *Ncf1^∗/∗^* animals when compared to B10.Q wild-type mice [13]. Mutated mice also developed arthritis quicker after the booster allowing us to work with a more homogenous and extremely well reproducible disease model. Better arthritis synchronization in the mutated mouse model together with larger treatment groups resulted in less variability and increased statistical power in data analysis. In proteose peptone-induced peritonitis we observed no difference between the genotypes. The use of *Ncf1* mutant mice allowed us to avoid pit falls associated with the use of chemical NOX2 complex inhibitors such as DPI and apocynin, which are not specific for the NOX2 complex, do not provide full suppression of superoxide production and additionally profoundly affect various other cellular processes [14–16].

 In both arthritis and peritonitis, adenoviral gene expression vectors locally enhanced inflammation. The arthritis enhancing effect was restricted to the treated paw, as the virus-injected groups did not differ from the PBS-injected control group when their nontreated paws were analyzed. This is in line with previous reports where intra-articular injections of adenoviral gene expression vectors have been shown to induce increased paw swelling and elevated levels of inflammation mediators [33, 34]. Intravenous injection route has not given rise to enhanced arthritis [35–37], but has triggered liver inflammation; liver being the primary target of systemically administered adenoviral vectors [35]. The immunogenicity of the adenoviral gene expression systems is well documented in the literature [38–40].

ROS regulate a number of physiological and disease-related pathways in humans. SOD3 polymorphisms are associated with COPD [41], coronary artery disease, myocardial infarction [42] as well as acute lung injury and related mortality [43]. Additionally, SOD3 has been reported to be downregulated in thyroid cancer tumors [44]. Both SOD3 and *Ncf1* are highly conserved ROS regulators [45]. There is also genetic [46] and functional [47] evidence linking *Ncf1* to human diseases.

## 5. Conclusions

We report that SOD3 limits inflammation in CIA and peritonitis both in the presence and in the absence of phagocyte oxidative burst. The anti-inflammatory function of SOD3 is not compromised by the lack of functionality of the NOX2 complex as both *Ncf1^+/+^* and *Ncf1^∗/∗^* mice develop milder inflammation when treated with SOD3. Thus, we conclude that the anti-inflammatory effect of SOD3 is not dependent on superoxide produced by the NOX2 complex derived phagocyte oxidative burst and thus acts via other signaling pathways. 

## Supplementary Material

Supplementary figure 1. The expression of SOD3 did not affect arthritis severity in the control paws. Arthritis severity in the non-treated control paws was not affected in either wild type (A) or *Ncf1*∗/∗ mice (B). Sum score of all three non-treated paws is presented for both genotypes. In wild type mice (Ade-SOD3 *n*=6, Ade-lacZ *n*=7) and in *Ncf1*∗/∗ mice (Ade-SOD3 *n*=22, Ade-lacZ *n*=23).
