# Supplementary material for: Superoxide Dismutase 3 Limits Collagen-Induced Arthritis in the Absence of Phagocyte Oxidative Burst
Source: Mediators Inflamm. 2012 Mar 5;2012:730469. doi: 10.1155/2012/730469 (PMC3317049; doi:10.1155/2012/730469)
Supplement: Supplementary file 1 — Supplementary figure 1. The expression of SOD3 did not affect arthritis severity in the control paws. Arthritis severity in the non-treated control paws was not affected in either wild type (A) or Ncf1∗/∗ mice (B). Sum score of all three non-treated paws is presented for both genotypes. In wild type mice (Ade-SOD3 n=6, Ade-lacZ n=7) and in Ncf1∗/∗ mice (Ade-SOD3 n=22, Ade-lacZ n=23). [file 730469.f1.pdf]

Supplementary figure 1. The expression of SOD3 did not affect arthritis severity in the control paws. Arthritis severity in the non-treated control paws was not affected in either wild type (A) or *Ncf1 */** mice (B). Sum score of all three non-treated paws is presented for both genotypes. In wild type mice (Ade-SOD3 n=6, Ade-lacZ n=7) and in *Ncf1*/** mice (Ade-SOD3 n=22, Ade-lacZ n=23).

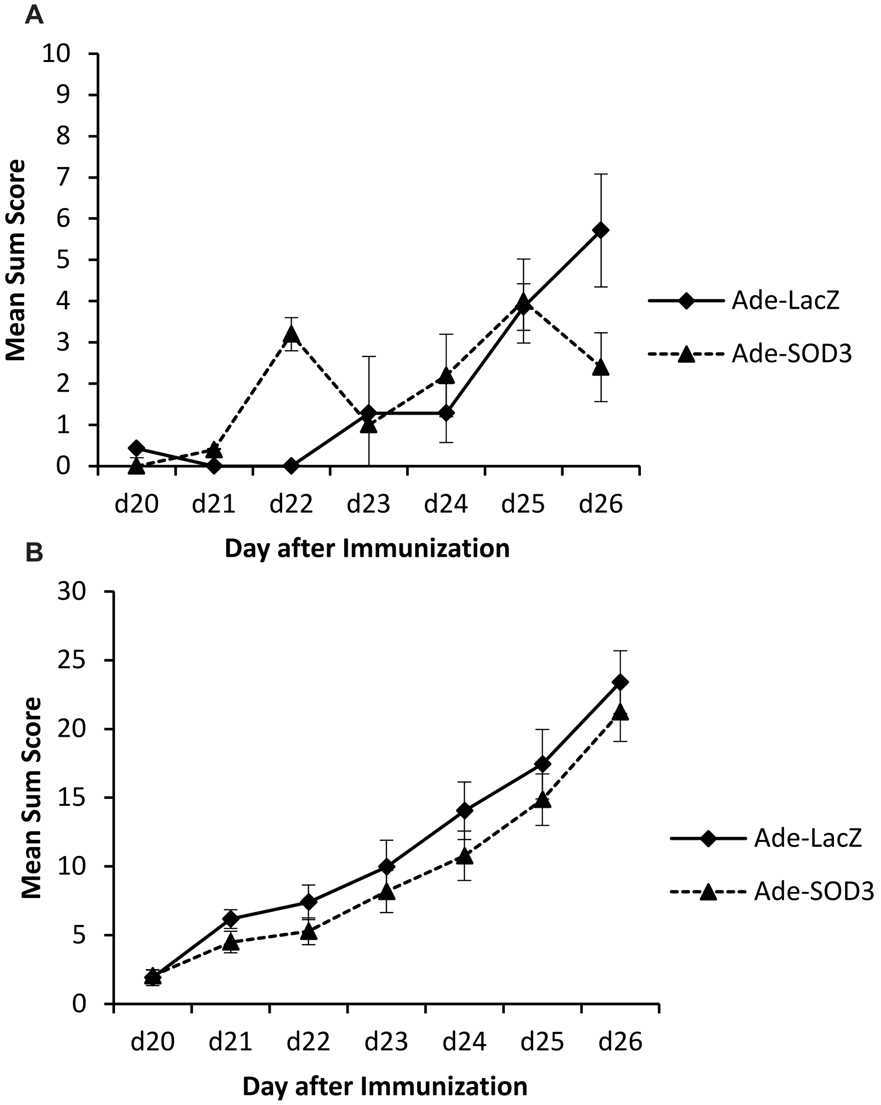

Supplementary figure 1
